# Intraperitoneal and subcutaneous glucagon delivery in anaesthetized pigs: effects on circulating glucagon and glucose levels

**DOI:** 10.1038/s41598-020-70813-5

**Published:** 2020-08-13

**Authors:** Marte Kierulf Åm, Ilze Dirnena-Fusini, Anders Lyngvi Fougner, Sven Magnus Carlsen, Sverre Christian Christiansen

**Affiliations:** 1 Department of Clinical and Molecular Medicine, Faculty of Medicine and Health Sciences, Norwegian University of Science and Technology (NTNU), Postboks 8905, 7491 Trondheim, Norway; 2 Department of Endocrinology, St Olav’s Hospital, Trondheim, Norway; 3 Department of Engineering Cybernetics, Faculty of Information Technology and Electrical Engineering, Norwegian University of Science and Technology (NTNU), Trondheim, Norway

**Keywords:** Endocrinology, Diabetes

## Abstract

Glucagon is a pancreatic hormone and increases the blood glucose levels. It may be incorporated in a dual hormone artificial pancreas, a device to automatically and continuously control blood glucose levels of individuals with diabetes. Artificial pancreas systems have been developed for use in the subcutaneous tissue; however, the systems are not fully automated due to slow dynamics. The intraperitoneal space is therefore investigated as an alternative location for an artificial pancreas. Glucose dynamics after subcutaneous and intraperitoneal glucagon delivery in ten anaesthetized pigs were investigated. The pigs received intraperitoneal boluses of 0.3 µg/kg and 0.6 µg/kg and a subcutaneous bolus of 0.6 µg/kg in randomized order. They also received an intraperitoneal bolus of 1 mg given at the end of the experiments to test the remaining capacity of rapid glucose release. Six pigs were included in the statistical analysis. The intraperitoneal glucagon bolus of 0.6 µg/kg gave a significantly higher glucose response from 14 to 30 min compared with the subcutaneous bolus. The results indicate that glucagon induces a larger glucose response after intraperitoneal delivery compared with subcutaneous delivery and is encouraging for the incorporation of glucagon in an intraperitoneal artificial pancreas.

## Introduction

An artificial pancreas (AP) is a device that automatically and continuously regulates insulin delivery to control the blood glucose level (BGL) in people with diabetes, primarily patients with diabetes mellitus type 1 (DM1). The AP system holds great promise for relieving the patients of the potentially serious side-effects of the disease and the everyday burden of diabetes management^1,2^. However, the full potential of the AP remains to be realized mainly due to the markedly delayed glucose lowering response of subcutaneous (SC) delivered insulin. Hence, the intraperitoneal (IP) space is investigated as an alternative location for hormone delivery in an AP.

Incorporating glucagon in the AP system, i.e. a dual hormone AP, might enable tighter glucose control^3^. At present, glucagon is mainly used as an emergency treatment to counteract severe, insulin-induced hypoglycaemia and is injected either SC or intramuscularly (IM). The prescribed dose of 1 mg is large and has a single goal of promptly elevating the BGL. In an AP, however, the aim of glucagon delivery is not primarily to treat hypoglycaemia but to prevent imminent hypoglycaemia by small, and possibly repeated, doses. Mini-doses of SC glucagon are efficient in treating milder hypoglycaemia in children as well as in adults, and the glucose rising effect is the same regardless of hypoglycaemic or euglycaemic BGL^4^. Studies have shown that repeated mini-doses of glucagon did not deplete the liver of glycogen and that the glucose increasing effect was similar regardless of previous boluses^4,5^. This supports the use of glucagon in a dual hormone AP.

Both single hormone (insulin only) and dual hormonal (insulin and glucagon) AP systems show benefits compared with insulin treatment by multiple daily injections or by a pump, especially during night-time^2,6^. Dual hormone AP shows further improvement compared with single hormone AP regarding time spent in the hypoglycaemic range and prevention of hypoglycaemic episodes, especially in relation to exercise^3^. In addition, dual hormone AP seems to reduce carbohydrate intake, as carbohydrates are often needed to correct mild hypoglycaemia^3^. Although the AP systems show improvement for patients, a fully automatic AP system is yet to be developed, as the only commercially available system still requires users to manage meal insulin boluses^7,8^. The slow dynamics of the SC tissue is probably the main obstacle that prevents these systems from being fully automated^9^. Moving the hormone delivery of an AP into the peritoneal space, can lead to faster absorption and improved glycaemic control, which has been shown for IP insulin delivery^10–12^. A large portion of IP injected insulin is transported by the portal vein directly to the liver, mimicking the normal physiological “first-pass effect”^12,13^. The faster and more appropriate insulin absorption is the main advantage of the IP AP system and adding glucagon as a second hormone in the IP AP system is only a minor adjustment and of interest even if the glucose response after IP delivery of glucagon would be similar to that of SC delivery.

IP glucagon delivery has only been investigated in a few animal studies^14–17^, and if glucagon is to be incorporated in a dual hormone IP AP, the kinetics of glucagon absorption and effect on BGLs need to be more closely identified. We have previously studied the glucose response after IP glucagon delivery in rats and found a faster effect compared with SC delivery^17^. The aim of the present study was to reproduce the experiment in another and more relevant animal model, and explore the glucose dynamics after IP delivery of glucagon and compare it to SC delivery.

## Results

Ten pigs received at least four glucagon boluses, except one pig, which only received two boluses (0.6 µg/kg SC and IP) due to circumstances unrelated to the experiment.

### Glucose response

No apparent elevation in BGL was observed in four of nine 0.3 µg/kg IP boluses, three of ten 0.6 µg/kg IP boluses, one of ten 0.6 µg/kg SC boluses, and four of nine 1 mg IP boluses. Neither of the two intravenous (IV) boluses elevated the BGL. Figures and tables in the article are therefore presenting the pigs which showed a glucose response of at least 1 mmol/L after the last 1 mg IP dose in addition to the pig which only received two boluses (n = 6). Results from all pigs (n = 10) are presented as Supplementary materials online.

**Figure 1:**
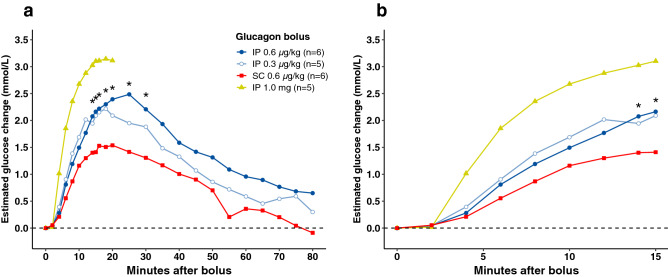
Glucose dynamics. Estimated glucose changes for the full 80 min (**a**) and for the first 15 min (**b**) after glucagon delivery in pigs, which responded with a BG rise after the 1 mg IP bolus. The 0.6 µg/kg IP bolus gave significantly higher glucose elevations compared with the 0.6 µg/kg SC bolus from time points 14–30 min (marked with *). 1 mg IP bolus gave significantly higher glucose elevations compared with all other boluses from 4 to 20 min, except at 20 min compared to the 0.6 µg/kg IP bolus (not marked in the graph).

**Figure 2:**
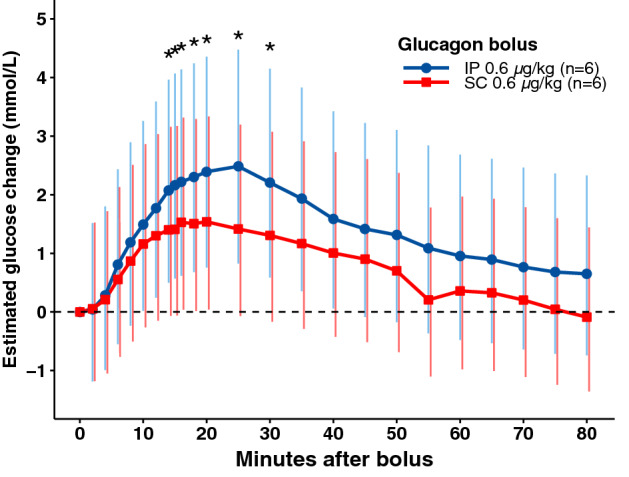
Glucose dynamics for the 0.6 µg/kg IP and 0.6 µg/kg SC bolus. Estimated glucose changes and 95% confidence intervals for the full 80 min after glucagon delivery in pigs, which responded with a BG rise after the 1 mg IP bolus. The 0.6 µg/kg IP bolus gave significantly higher glucose elevations compared with the 0.6 µg/kg SC bolus from time points 14–30 min (marked with *). The p-values for these time points were 0.033, 0.022, 0.042, 0.027, 0.023, 0.009, and 0.037, respectively.

The estimated blood glucose (BG) changes from baseline, estimated by a mixed linear model for the different boluses, are displayed in Fig. 1. The 0.6 µg/kg IP bolus gave significantly higher glucose elevations compared with the 0.6 µg/kg SC bolus at time points between 14 and 30 min (Fig. 2) The p-values are reported individually in the figure legend. The 1 mg IP bolus gave significantly higher glucose elevations compared with all other boluses from 4 to 20 min (p < 0.05) except at 20 min compared to the 0.6 µg/kg IP bolus. We found no statistically significant differences comparing the 0.3 µg/kg IP and the 0.6 µg/kg SC bolus (Supplementary Table S3 online). When including all ten pigs in the analyses, we found no statistically significant differences in BG elevations between any of the different boluses for any of the different time points (Supplementary Fig. S1 and Table S4 online). No statistically significant differences in AUC or time to maximum glucose response were found when comparing the different boluses (Table 1 and Supplementary Table S1 online).

**Table 1:** Pharmacodynamics and kinetics of IP and SC glucagon boluses.

	Glucose				Glucagon		
	N^a^	CΔ_max_ ± SD (mmol/l)	T_max_ ± SD (min)	AUC_0-80 min_ ± SD (mmol/l x min)	n^a^	C_max_ ± SD (pmol/L)	T_max_ ± SD (min)
0.6 µg/kg IP	6	2.4 ± 1.0	22.2 ± 4.5	101 ± 53	5	66.0 ± 62.0	13.3 ± 7.2
0.6 µg/kg SC	6	1.6 ± 0.9	19.3 ± 5.7	54 ± 47	3	23.3 ± 4.6	6.0 ± 2.0
0.3 µg/kg IP	5	2.2 ± 1.7	20.0 ± 7.4	92 ± 90	4	27.3 ± 19.7	38.5 ± 29.6

Data are arithmetic means ± standard deviations (SD).

*CΔ*_*max*_ maximum plasma concentration change, *C*_*max*_ maximum plasma concentration, *T*_*max*_ time to maximum plasma concentration.

^a^Samples for glucagon and insulin analysis were not collected from all boluses. For that reason, the numbers of included boluses differ between glucose and glucagon results.

**Figure 3:**
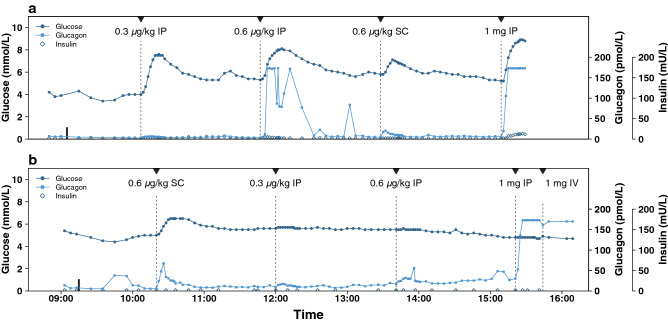
Glucose, insulin, and glucagon profiles from two full experiments. Glucagon boluses are marked with triangles and vertical dashed lines. The top graph shows pig no. 10, which responded with a rise in BG values after every glucagon bolus (**a**), while the bottom graph shows pig no. 11, which only responded to the first glucagon bolus although high levels of glucagon were detected in peripheral blood after all boluses (**b**). Only the first octreotide and pasireotide treatments are marked as vertical solid bars around 9 o’clock. Please see the methods section for full description.

Figure 3 displays the experiments on two different pigs illustrating the difference in glucose responsiveness. Figure 3a shows pig no. 10, which responded with BG increase after all four glucagon boluses. The glucose rise was approximately 2.2 and 3.7 mmol/L after the 0.6 µg/kg and 0.3 µg/kg IP boluses, respectively, and 1.3 mmol/L after the SC bolus. Figure 3b shows pig no. 11, which responded to the first glucagon bolus with a BG increase of 1.5 mmol/L, but did not show apparent glucose responses to the subsequent three boluses despite large measured glucagon levels in the circulation. Pig no. 11 also received a 1 mg IV glucagon bolus at the end of the experiment without any detectable glucose response.

### Glucagon absorption

Elevated peripheral blood glucagon concentrations were detected after 13 of 18 IP boluses and all five SC boluses from which blood samples were available for analysis. However, only 14 of these doses resulted in glucose elevations larger than 1 mmol/L. Mean maximum glucagon levels and mean time to maximum value for the different boluses for the included pigs are displayed in Table 1, and for all pigs in Supplementary Table S1 online. Glucose and glucagon curves from two experiments are shown in Fig. 3. The maximum detection limit for the ELISA kits was 170 pmol/L and was reached in both pig 10 and 11 (Fig. 3a,b).

### Endogenous hormone production

Endogenous insulin was detected in five of the six included pigs. The maximum concentration detected in these individual pigs were 3.5, 6.6, 7.6, 11.3, and 13.3 mU/L. Of the four excluded pigs, only one pig had detectable levels of endogenous insulin with a maximum concentration of 4.5 mU/L. Figure 3 presents the endogenous insulin concentrations obtained from two individual pigs throughout the one-day experiments. The mean ± SD glucagon and insulin concentrations before and after the first somatostatin analogue treatment are presented in Supplementary Table S2 online.

## Discussion

This study indicates that IP delivery of glucagon induces a larger glucose response after 14–30 min compared with SC delivery of the same glucagon dose (0.6 µg/kg) in somatostatin analogue treated pigs. This indicates a faster glucose increasing effect by the IP route compared with the SC route. However, the time to maximal BG response is observed around 20 min regardless of dose or route of delivery (Table 1 and Supplementary Table S1). This is in line with previously published results for SC delivery (15–20 min)^4, 18,19^.

IP glucagon kinetics and dynamics have only been studied in a few animal experiments^14–17^. Most previously published studies measured glucose only at a few time points, showing that IP glucagon delivery increase BGL but not documenting the full glucose response. We previously conducted a study in rats with IP glucagon delivery and frequent blood sampling, and showed that the glucose response after IP delivery was significantly higher after 4 min compared with SC delivery, indicating a faster glucose response after IP delivered glucagon^17^. The glucose elevation was also of shorter duration after IP delivery of glucagon. In the present study, we found a significantly larger glucose increase from 14 to 30 min after IP glucagon delivery, but we did not observe a faster initial glucose response, an earlier glucose peak, or a shorter duration of the glucose response compared with SC delivery of glucagon. This underlines the relevance of repeating our previous IP glucagon study in another animal model considered more closely resembling human physiology.

The peritoneal lining consists of a monolayer of mesothelial cells that covers a layer of connective tissue containing several different cell types, blood and lymph vessels^20,21^. The peritoneal lining is permeable to water, small solutes, and proteins^22^ and after IP delivery, a portion of the glucagon will diffuse through the visceral peritoneum and be transported directly to the liver via the portal vein. This route of drug delivery has been described for insulin showing a fast and efficient absorption to the portal vein and a fast effect on the BGL^23,24^. We hypothesize that glucagon delivered by the IP route reaches the liver earlier and at a high concentration and induces a fast and large glucose response because the IP route resembles normal glucagon secretion, i.e. from the pancreas and directly into the portal vein. This fits with our observation of a large glucose increasing effect even with the smallest IP dose (0.3 µg/kg), while hardly any increase in circulating glucagon levels was observed (Fig. 3 a). Similar glucose elevations were also observed after the 0.6 µg/kg and 1 mg IP boluses but with concomitant significant increased levels of glucagon in the systemic circulation. The concentration of glucagon in the blood reaching the liver after SC administration will be equivalent to what is reaching the rest of the body, and high concentrations of glucagon in the peripheral circulation can lead to unwanted side effects, such as nausea, vomiting, and increased heart rate.

In an AP system, glucagon will primarily be administered to avoid, not treat, hypoglycaemia. Hence, we focused on glucagon doses that would elevate the BGL by one to three mmol/L. Small SC doses of glucagon to treat mild hypoglycaemia have been studied before in both children^25,26^and adults^18,27,28^ with DM1 and in streptozotocin treated pigs^29^. Small doses of glucagon have also been studied in the context of an SC AP system^4,5,30,31^. There is no common definition of a “mini-dose” of glucagon or a consensus of optimal glucose response. Hence, the study protocols vary making comparisons between available data challenging. There is a dose–response relationship between glucagon and glucose response after SC delivery, with larger doses of glucagon resulting in higher glucose elevations^4,18,30^. However, this dose–response relation seems only to apply to smaller doses of glucagon and increasing doses above a certain level will not result in higher glucose responses^4,18,32^. This is in line with our results where the glucose elevations after the 1 mg IP bolus was only 0.5 mmol/L larger than the glucose elevation after the 0.3 µg/kg IP, even though the former dose is 80 times greater for a pig of 40 kg. It is acknowledged that glucagon binds to liver cells causing a non-linear increase of intracellular cAMP, where the half-maximum effect is reached already when 10% of the receptors are bound^33^.

We observed a larger variation in blood glucagon concentrations and glucose responses after IP delivery compared with SC delivery of glucagon (Fig. 2). This is in line with previously published results^14,17^ and might be related to varying amounts of IP fluid. We also observed large variation in glucose responses both between pigs and between boluses in the same pig regardless of route of delivery. This might, to some extent, be explained by differences in available liver glycogen. Some pigs in our study responded poorly or not at all to glucagon, even though glucagon was detected in peripheral blood (Fig. 3b). El Khatib et al. conducted a study on streptozotocin treated pigs that were fasted for 20 h before receiving a single dose of SC glucagon, but in contrast to our study these pigs had elevated fasting BGLs and received an initial insulin bolus and consequently a drop in BGLs^29^. Prolonged fasting has been shown to reduce the amount of available liver glycogen in humans after an overnight fast^5^ and in pigs before slaughter^34^. Repeated glucagon boluses have not shown any statistically significant depletion of liver glycogen or less effect of the final glucagon boluses in people with DM1^4,5^. Contrasting results, however, were observed in healthy volunteers where the second glucagon dose showed a marked lower effect compared with the first of two repeated glucagon doses of 0.5 mg^35^. In the present study, we observed no statistically significant differences in glucose response comparing the different order of bolus. However, Fig. 3b shows a pig responding to the first bolus but not to the later ones even though high levels of glucagon were detected in plasma. This lack of glucose response suggests a depletion of hepatic glycogen stores available for fast glucose production by glycogenolysis in some of the pigs. A possible explanation of the depletion of glycogen storage is prolonged fasting and repeated glucagon boluses. Hepatic glycogen depletion has been considered a potential, but not crucial, barrier for a successful dual hormone AP system in human trials^31,36^, and is a factor to be considered when studying glucagon dynamics using animal models.

The strength of the present study is frequent blood sampling and identification of the glucagon kinetics and dynamics during and after IP glucagon boluses, which enables the development of algorithms for an IP dual hormone AP system. The study has, however, also several limitations. Even with repeated glucagon boluses in each pig the sample size of ten pigs is small. The absent glucose increasing effect in four pigs reduced the number of valid pigs in the study even further.

Experiments under anaesthesia will inevitably affect the pig’s physiology and influence the data. Prolonged IV infusion of fluids can lead to increased production of IP fluid in pigs^37,38^. The IP space can potentially hold several litres, while under normal conditions it contains only small amounts of IP fluid^39–42^. Delivering glucagon into large volumes of IP fluid would dilute the glucagon and slow down the absorption and effect. We restricted the amount of infused IV fluid during the experiments to minimize the accumulation of IP fluid and observed only a moderate increase in the volume. Still, we believe that increased amounts of IP fluid may have influenced the results and expect accumulation of peritoneal fluid to be a lesser problem during long-term, free-living settings, both in animals and in humans.

The IP catheter was placed in the upper left quadrant of the abdomen. As the tip of the catheter was free-moving, the exact site for glucagon delivery is unknown, but this approach is equivalent to the current solution for clinical IP insulin delivery by Accu-Check DiaPort (Roche) in humans. The absorption of glucagon over the peritoneal lining might differ throughout the IP space. We are not aware of any published data related to this topic.

We measured small amounts of porcine insulin in some of the pigs, indicating that endogenous insulin secretion was not completely suppressed. However, the levels detected are low compared with insulin levels of naive pigs undergoing oral and IV glucose tolerance tests^43^. In the present study somatostatin analogues seemed to suppress endogenous glucagon secretion, as the concentration of glucagon was lowered after the initial somatostatin analogue administration. We observed some fluctuations in the measured glucagon level within the same time series, which we cannot explain (e.g. example in Fig. 3a).

In conclusion, glucagon gives a larger rise in BGL when delivered IP compared with SC in somatostatin analogue treated pigs under prolonged anaesthesia. These results imply that smaller doses of glucagon can be given IP with the same glucose increasing effect, potentially avoiding unwanted side effects of glucagon treatment.

## Materials and methods

### Animals and animal handling

Between February and June 2018, eleven female, juvenile, non-diabetic farm pigs (*Sus scrofa*), weighing 34–50 kg, were brought to the animal research facility approximately one week before experiments and acclimated to the staff and new environment. They were housed together in a common pen, in groups of two or three whenever possible, provided wood chips as nesting material and toys to keep them occupied. The lighting condition was standardized with a 16 h light period followed by an 8 h dark period. They were fed standard commercial growth feed twice a day and provided water ad libitum. Food was removed nine-ten hours prior to the experiments.

### Anaesthesia

The pigs were premedicated with an IM injection of 4 mg diazepam (Stesolid^®^, Actavis Group, Hafnarfjordur, Iceland), 160 mg azaperone (Stresnil^®^, Eli Lilly Regional Operations GmbH, Austria) and 750 mg ketamine (Ketalar^®^, Pfizer AS, Norway), while in the pen. An ear vein was cannulated, and anaesthesia was induced with an IV injection of 1 mg atropine (Takeda AS, Asker, Norway), 150–250 µg fentanyl (Actavis Group, Hafnarfjordur, Iceland), 75–125 mg thiopental (VUAB Pharma AS, Roztoky, Czech Republic) and 150–250 mg ketamine (Ketalar^®^, Pfizer AS, Norway).

The pigs were intubated in the lateral position and mechanically ventilated and monitored on an anaesthesia machine (Aisys, GE Healthcare Technologies, Oslo). Anaesthesia was maintained by IV infusion of midazolam (0.5 mg/kg/h) (Accord Healthcare Limited, Middlesex, UK) and fentanyl (7.5 µg/kg/h) (Actavis Group, Hafnarfjordur, Iceland) and by inhalation of isoflurane (0–2%) (Baxter AS, Oslo, Norway). The room temperature was around 20 °C. The body temperature of the pigs was continuously monitored, and a heating blanket applied when necessary.

The pigs received IV infusions of antibiotics (Cefalotin, Villerton Invest SA, Luxembourg), 2 g immediately after the pigs were anaesthetized and 1 g after 4 h. Heparin (150 IE) (LEO Pharma A/S, Ballerup, Denmark) was injected in the peritoneal space at the same time points.

Fluid balance was achieved by continuous IV infusion of Ringer’s acetate, approximately 1,000 ml during the length of the experiment, with individual adjustments. The described anaesthesia protocol have been used in previous pig experiments^37^.

### Surgical procedure

An intra-arterial line was placed in the left carotid artery for blood sampling and monitoring of physiological parameters and an IV line was placed in the left internal jugular vein for fluid infusions. Both catheters were inserted through the same cut-down.

The catheter from an Animas Vibe insulin pump (serial number 80-26400-16, Animas Corp., West Chester, PA, USA) was inserted 10–15 cm into the upper left part of the abdomen through a 2–3 cm long craniocaudal incision in the abdominal wall, 2–3 cm caudally to the umbilicus. The bladder was exposed through a small, low laparotomy for the insertion of a bladder catheter. Both cuts were made with a thermocauter to minimize bleeding.

At the end of the experiments, and under full anaesthesia, the pigs were euthanised with an IV overdose of pentobarbital (minimum 100 mg/kg) (pentobarbital NAF, Apotek, Lørenskog, Norway).

### Suppression of endogenous glucagon secretion

To inhibit endogenous insulin and glucagon secretion, the pigs were given IV injections of 0.4 mg octreotide (Sandostatin 200 µg/ml, Novartis Europharm Limited, United Kingdom) every hour and SC injections of 0.3 mg pasireotide (Signifor 0.3 mg/ml, Novartis Europharm Limited, United Kingdom) every third hour. To verify glycaemic stability, three blood samples were collected within the last 20 min before the first injection of somatostatin analogues. Suppression efficiency was evaluated by analysing glucagon and insulin levels for 30 min after somatostatin analogue injection (every 10 min). Blood samples were also analysed for porcine insulin at intervals throughout the experiments.

The effectiveness of the somatostatin analogues for the suppression of glucagon and insulin secretion is described in the last paragraph of the Results section and in Supplementary Table S2.

### Glucagon boluses

Glucagon (Novo Nordisk, Denmark) was mixed according to specification and placed in the pump. The volume of one unit by the pump was measured to 10 µl, and 1 unit was equivalent to 10 µg of glucagon. The glucagon in the pump was stored at room temperature for the length of the one-day experiments. Glucagon for the SC dose and the last 1 mg IP dose were mixed just before administration.

One pig was used to define the doses of glucagon to be used in the experimental protocol and not included in the analysis. We aimed at boluses which would raise the BGL with one to three mmol/L and chose to investigate three different IP doses of glucagon (0.3 µg/kg, 0.6 µg/kg and 1 mg) and one SC dose (0.6 µg/kg). The 0.3 µg/kg IP, 0.6 µg/kg IP and 0.6 µg/kg SC doses were delivered in a randomized order, with approximately 100 min between each bolus, while the 1 mg IP bolus was given as the last bolus to test maximum glucose effect of IP glucagon delivery. Two pigs, which did not show any post glucagon elevations in BGL, received a 1 mg IV dose at the end of experiments.

### Glucose analysis

Arterial blood samples for glucose analysis were collected every 2–15 min throughout the length of experiments, with the highest frequency from 15 before to 80 min after glucagon boluses. Samples were collected in heparinized syringes (LEO Pharma A/S, Ballerup, Denmark) and analysed on a Radiometer ABL 725 blood gas analyser (Radiometer Medical ApS, Brønshøj, Denmark). Most samples were analysed immediately, but some samples were stored on ice for a maximum of 20 min before analysis. We have previously published that such samples can be stored on ice for up to six hours without significant effect on measured glucose values^37^.

### Glucagon and porcine insulin analysis

Three arterial blood samples within the last 20 min before glucagon boluses were analysed for glucagon and insulin in all pigs. However, arterial blood samples, with the same intervals as glucose samples described above, were only collected from 8 pigs, in total after nine IP boluses and four SC boluses. Arterial blood samples were collected in empty syringes and immediately transferred to EDTA vacutainers (2 ml). The samples were stored in ice water for 10 min before they were centrifuged and the plasma transferred to Eppendorf tubes and stored at − 80 °C until analysis. Glucagon was analysed with Glucagon ELISA (10-1281-01 Mercodia, Uppsala, Sweden) and porcine insulin was analysed with Porcine Insulin ELISA (10-1200-01, Mercodia, Uppsala, Sweden). The assay ranges for the glucagon and porcine insulin ELISA kits were 2–172 pmol/L and 2.3–173 mU/L, respectively.

All glucagon samples were run in singles with a coefficient of variability (CV) < 10%. Inter-assay CV were 8%, 8% and 6% for 42.6, 14.7 and 4.98 pmol/L standards, respectively. This glucagon ELISA kit cannot differentiate between endogenous and exogenous glucagon.

### Statistical analysis

The relationship between BGL and time was analysed for all interventions using a mixed linear model with bolus order and the combination of time and treatments as the fixed effects. The dependent variable was defined as log BGL to achieve normal distribution. To account for multiple measurement series on each pig, pig identification was included as a random effect^44^. To account for dependence within each series, the error term for each series was specified as a first-order autoregressive process (AR (1)) series accounting for minutes between measurements. The estimated mean changes in BGL, from the mixed linear model, for time points 0–80 min for the 0.6 µg/kg IP, 0.3 µg/kg IP and 0.6 µg/kg SC boluses and time points 0–20 min for the 1 mg IP bolus were compared using the Wald test. The area under the curve (AUC) for delta values from minute 0 to 80 and time until maximum BG change for the different boluses were compared using the Mann–Whitney U test. If the maximum value for BG change was observed for more than one time point, the first occurring time point was selected. The level of significance was set to 0.05. All statistical analyses were carried out in R^45^.

### Ethical approval

The animal experiments were approved by the Norwegian Food Safety Authority (FOTS number 12948) and were in accordance with “The Norwegian Regulation on Animal Experimentation” and “Directive 2010/63/EU on the protection of animals used for scientific purposes”.

## Supplementary information

Supplementary information

## Data Availability

Additional data is available as supplementary material.
