# Supplementary material for: Intraperitoneal and subcutaneous glucagon delivery in anaesthetized pigs: effects on circulating glucagon and glucose levels
Source: Sci Rep. 2020 Aug 13;10:13735. doi: 10.1038/s41598-020-70813-5 (PMC7426268; doi:10.1038/s41598-020-70813-5)
Supplement: Supplementary file 1 — Supplementary information [file 41598_2020_70813_MOESM1_ESM.pdf]

Intraperitoneal and subcutaneous glucagon delivery in anaesthetized pigs: Effects on circulating glucagon and glucose levels

Marte Kierulf Åm, Ilze Dirnena–Fusini, Anders Lyngvi Fougner, Sven Magnus Carlsen, Sverre Christian Christiansen

Supplementary table S1: Characteristics of all and included pigs before and after the first treatment with somatostatin analogues.

Characteristics	All pigs (n=10)	Included pigs (n=6)
Body weight (mean ± SD, kg)	42.6 ± 5.8	43.9 ± 5.6
Sex (% female)	100	
Blood glucose **before** * (mean ± SD, mmol/L)	4.7 ± 1.0	5.0 ± 0.8
Blood glucose **after** * (mean ± SD, mmol/L)	4,7 ± 1.3	5.1 ± 0.9
Insulin **before** * (mean ± SD, mU/L)	2.7 ± 1.1	2.9 ± 1.4
Insulin **after** * (mean ± SD, mU/L)	2.5 ± 0.6	2.6 ± 1.8
Glucagon **before** * (mean ± SD, pmol/L)	14.4 ± 19.2	17.6 ± 25.1
Glucagon **after** * (mean ± SD, pmol/L)	8.0 ± 6.2	7.9 ± 7.1

*** first somatostatin analogue treatment. SD = standard deviation.**

Supplementary table S2: Pharmacodynamics of IP and SC glucagon boluses for all pigs in the study.

	Glucose				Glucagon		
**Glucagon bolus**	**n†**	**$C\Delta_{max}$ ± SD (mmol/l)**	**T_{max} ± SD (min)**	**$AUC_{0–80\ min}$ ± SD (mmol/l x min)**	**n†**	**C_{max} ± SD (pmol/L)**	**T_{max} ± SD (min)**
0.6 µg/kg IP	10	1.7 ± 1.3	21.2 ± 12.4	75 ± 59	8	60.2 ± 47.6	13.1 ± 6.6
0.6 µg/kg SC	10	1.2 ± 0.9	15.5 ± 7.6	43 ± 43	5	34.0 ± 19.5	6.0 ± 1.4
0.3 µg/kg IP	9	1.5 ± 1.5	28.8 ± 29.2	59 ± 76	6	22.97 ± 16.7	40.0 ± 32.9

Abbreviations: $C\Delta_{max}$, maximum plasma concentration change; C_{max}, maximum plasma concentration; T_{max}, time to maximum plasma concentration. Data are arithmetic means ± standard deviations (SD).
(†) Samples for glucagon and insulin analysis were not collected from all boluses. For that reason, the numbers of boluses differ between glucose and glucagon results.

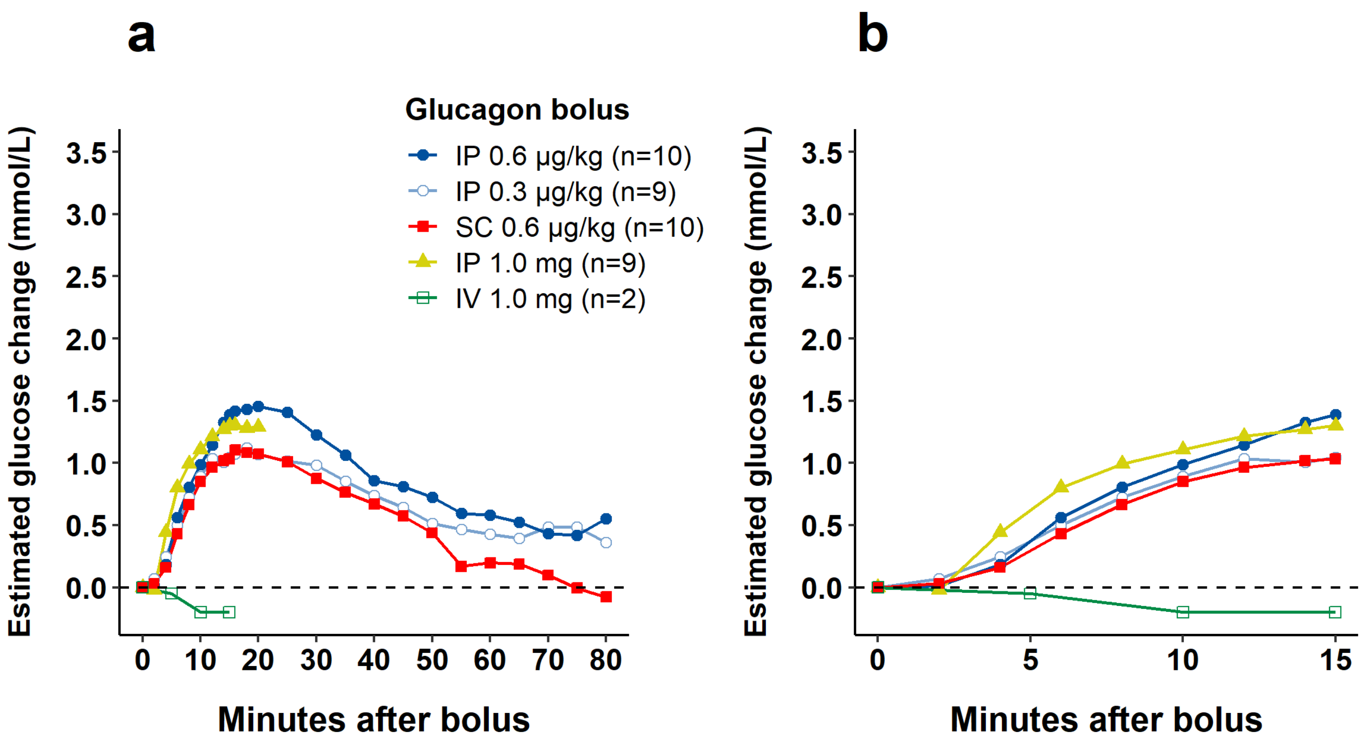

Supplementary figure S1: Glucose dynamics. Estimated glucose changes for the full 80 minutes (a) and for the first 15 minutes (b) after glucagon delivery in all pigs for all boluses.

Supplementary table S3: Estimated glucose elevations with 95% confidence intervals of included pigs.

Minutes	IP 06 µg/kg (n=6)	SC 06 µg/kg (n=6)	IP 03 µg/kg (n=5)	IP 1 mg (n=5)
**2**	0.04 (-1.19 – 1.52)	0.05 (-1.18 – 1.52)	0.05 (-1.19 – 1.52)	0.02 (-1.22 – 1.49)
**4**	0.28 (-0.99 – 1.80)	0.21 (-1.05 – 1.72)	0.39 (-0.90 – 1.94)	1.01 (-0.39 – 2.70) **
**6**	0.81 (-0.55 – 2.43)	0.55 (-0.77 – 2.13)	0.90 (-0.47 – 2.55)	1.85 (0.30 – 3.71) **
**8**	1.19 (-0.23 – 2.89)	0.87 (-0.50 – 2.51)	1.38 (-0.07 – 3.13)	2.36 (0.71 – 4.33) **
**10**	1.49 (0.02 – 3.26)	1.16 (-0.26 – 2.86)	1.69 (0.18 – 3.50)	2.68 (0.97 – 4.73) **
**12**	1.77 (0.24 – 3.59)	1.30 (-0.15 – 3.03)	2.02 (0.45 – 3.89)	2.88 (1.13 – 4.99) **
**14**	2.08 (0.50 – 3.96) *	1.40 (-0.07 – 3.16) *	1.94 (0.39 – 3.81)	3.03 (1.24 – 5.18) **
**15**	2.16 (0.57 – 4.07) *	1.41 (-0.06 – 3.17) *	2.09 (0.51 – 3.99)	3.10 (1.30 – 5.28) **
**16**	2.22 (0.62 – 4.14) *	1.53 (0.04 – 3.32) *	2.15 (0.56 – 4.07)	3.11 (1.30 – 5.30) **
**18**	2.30 (0.68 – 4.24) *	1.51 (0.02 – 3.29) *	2.22 (0.61 – 4.15)	3.14 (1.31 – 5.36) **
**20**	2.39 (0.76 – 4.36) *	1.54 (0.04 – 3.33) *	2.09 (0.50 – 4.00)	3.11 (1.28 – 5.33) **
**25**	2.48 (0.83 – 4.47) *	1.42 (-0.07 – 3.20) *	1.95 (0.37 – 3.84)	
**30**	2.21 (0.59 – 4.15) *	1.31 (-0.17 – 3.07) *	1.88 (0.31 – 3.77)	
**35**	1.93 (0.35 – 3.83)	1.17 (-0.29 – 2.91)	1.48 (-0.03 – 3.30)	
**40**	1.59 (0.06 – 3.42)	1.01 (-0.43 – 2.73)	1.33 (-0.16 – 3.12)	
**45**	1.42 (-0.09 – 3.22)	0.90 (-0.52 – 2.61)	1.07 (-0.38 – 2.82)	
**50**	1.31 (-0.18 – 3.11)	0.70 (-0.69 – 2.37)	0.86 (-0.56 – 2.57)	
**55**	1.09 (-0.37 – 2.84)	0.21 (-1.10 – 1.78)	0.72 (-0.68 – 2.41)	
**60**	0.96 (-0.48 – 2.68)	0.36 (-0.98 – 1.97)	0.59 (-0.79 – 2.25)	
**65**	0.89 (-0.53 – 2.61)	0.33 (-1,01 – 1.93)	0.46 (-0.91 – 2.10)	
**70**	0.77 (-0.64 – 2.46)	0.20 (-1.11 – 1.79)	0.54 (-0.83 – 2.21)	
**75**	0.68 (-0.71 – 2.36)	0.04 (-1.24 – 1.60)	0.59 (-0.80 – 2.26)	
**80**	0.65 (-0.74 – 2.33)	-0.09 (-1.36 – 1.44)	0.30 (-1.04 – 1.92)	

*** Statistical significant difference. See Fig. 2 in the main text for individual p-values.**

**** IP 1 mg was significantly higher than all the other boluses from time points 4 to 20 minutes (p-value <0.05) except compared to 0.6 µg/kg IP at time point 20 minutes.**

Supplementary table S4: Estimated glucose elevations with 95% confidence intervals of all pigs.

Minutes	IP 06 µg/kg (n=10)	SC 06 µg/kg (n=10)	IP 03 µg/kg (n=9)	IP 1 mg (n=9)
**2**	0.01 (-1.23 – 1.57)	0.03 (-1.21 – 1.59)	0.07 (-1.18 – 1.64)	-0.02 (-1.25 – 1.53)
**4**	0.18 (-1.09 – 1.78)	0.16 (-1.11 – 1.75)	0.25 (-1.04 – 1.86)	0.44 (-0.89 – 2.11)
**6**	0.56 (-0.78 – 2.25)	0.43 (-0.89 – 2.09)	0.50 (-0.84 – 2.18)	0.80 (-0.60 – 2.56)
**8**	0.81 (-0.59 – 2.56)	0.67 (-0.70 – 2.39)	0.73 (-0.65 – 2.46)	0.99 (-0.45 – 2.81)
**10**	0.99 (-0.44 – 2.79)	0.85 (-0.55 – 2.62)	0.89 (-0.52 – 2.67)	1.11 (-0.36 – 2.96)
**12**	1.14 (-0.32 – 2.98)	0.96 (-0.46 – 2.76)	1.04 (-0.40 – 2.85)	1.22 (-0.28 – 3.09)
**14**	1.32 (-0.17 – 3.21)	1.02 (-0.42 – 2.83)	1.01 (-0.43 – 2.82)	1.27 (-0.23 – 3.17)
**15**	1.39 (-0.12 – 3.29)	1.03 (-0.41 – 2.85)	1.05 (-0.40 – 2.86)	1.30 (-0.21 – 3.21)
**16**	1.42 (-0.10 – 3.32)	1.10 (-0.35 – 2.94)	1.07 (-0.38 – 2.90)	1.31 (-0.21 – 3.22)
**18**	1.43 (-0.09 – 3.34)	1.08 (-0.37 – 2.91)	1.12 (-0.34 – 2.96)	1.28 (-0.23 – 3.19)
**20**	1.46 (-0.07 – 3.37)	1.07 (-0.38 – 2.90)	1.07 (-0.38 – 2.90)	1.29 (-0.23 – 3.21)
**25**	1.41 (-0.11 – 3.32)	1.01 (-0.43 – 2.83)	1.01 (-0.43 – 2.83)	
**30**	1.23 (-0.26 – 3.10)	0.88 (-0.54 – 2.67)	0.98 (-0.46 – 2.79)	
**35**	1.06 (-0.39 – 2.90)	0.76 (-0.64 – 2.53)	0.86 (-0.56 – 2.64)	
**40**	0.86 (-0.56 – 2.64)	0.67 (-0.71 – 2.42)	0.74 (-0.66 – 2.50)	
**45**	0.81 (-0.60 – 2.59)	0.57 (-0.79 – 2.30)	0.64 (-0.74 – 2.38)	
**50**	0.72 (-0.67 – 2.48)	0.44 (-0.91 – 2.13)	0.51 (-0.84 – 2.22)	
**55**	0.59 (-0.78 – 2.32)	0.17 (-1.12 – 1.80)	0.47 (-0.88 – 2.17)	
**60**	0.58 (-0.79 – 2.31)	0.20 (-1.10 – 1.83)	0.43 (-0.92 – 2.12)	
**65**	0.52 (-0.84 – 2.24)	0.19 (-1.11 – 1.82)	0.39 (-0.94 – 2.08)	
**70**	0.43 (-0.91 – 2.12)	0.10 (-1.18 – 1.72)	0.48 (-0.87 – 2.19)	
**75**	0.42 (-0.92 – 2.11)	-0.01 (-1.27 – 1.59)	0.48 (-0.87 – 2.20)	
**80**	0.55 (-0.81 – 2.27)	-0.08 (-1.33 – 1.50)	0.36 (-0.97 – 2.04)	
